# Supplementary figures and images for: Microbiota Composition and Evenness Predict Survival Rate of Oysters Confronted to Pacific Oyster Mortality Syndrome
Source: Front Microbiol. 2020 Feb 27;11:311. doi: 10.3389/fmicb.2020.00311 (PMC7056673; doi:10.3389/fmicb.2020.00311)

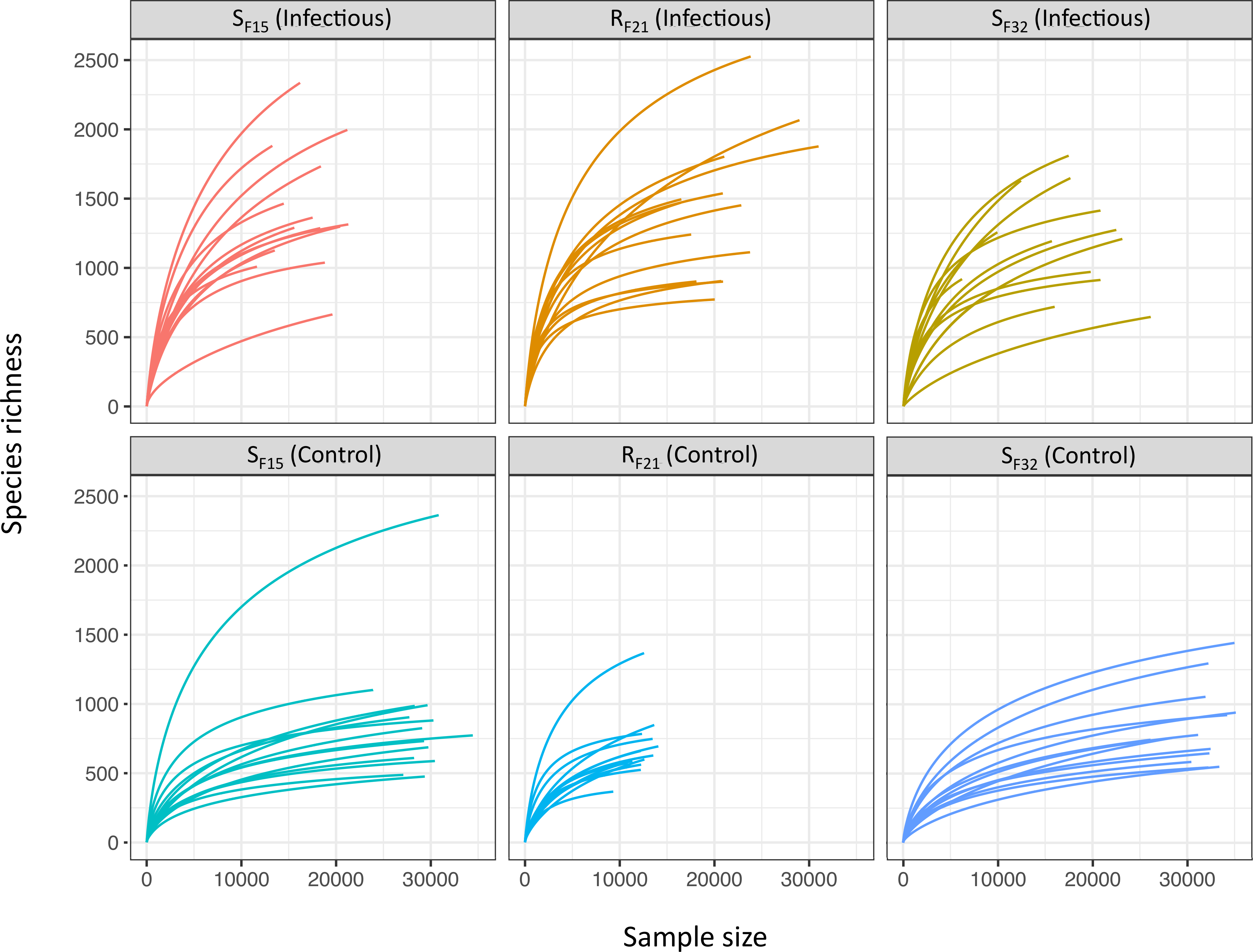

Supplement: Supplementary file 1 [file Image_1.TIFF]

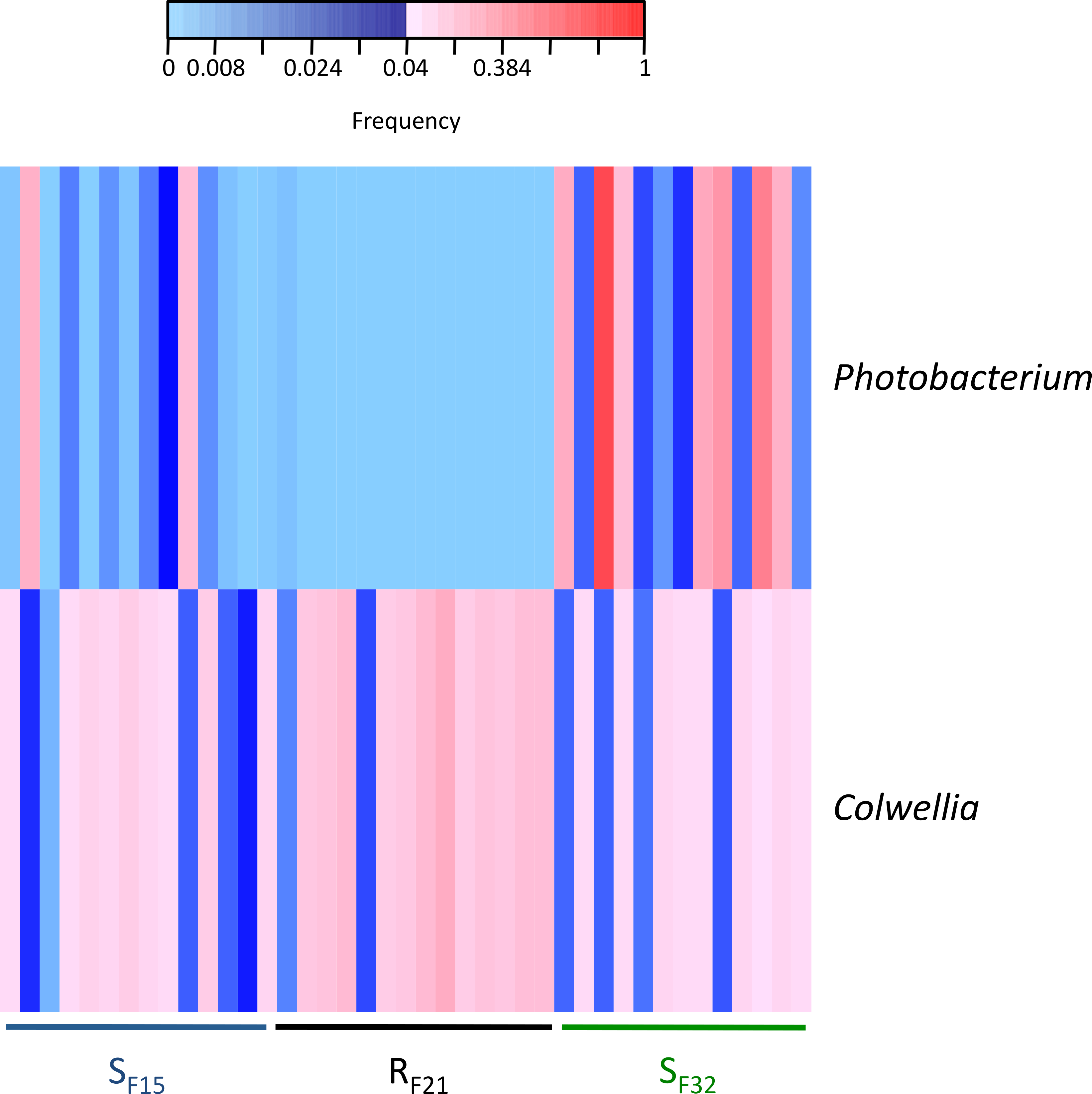

Supplement: Supplementary file 2 [file Image_2.TIFF]

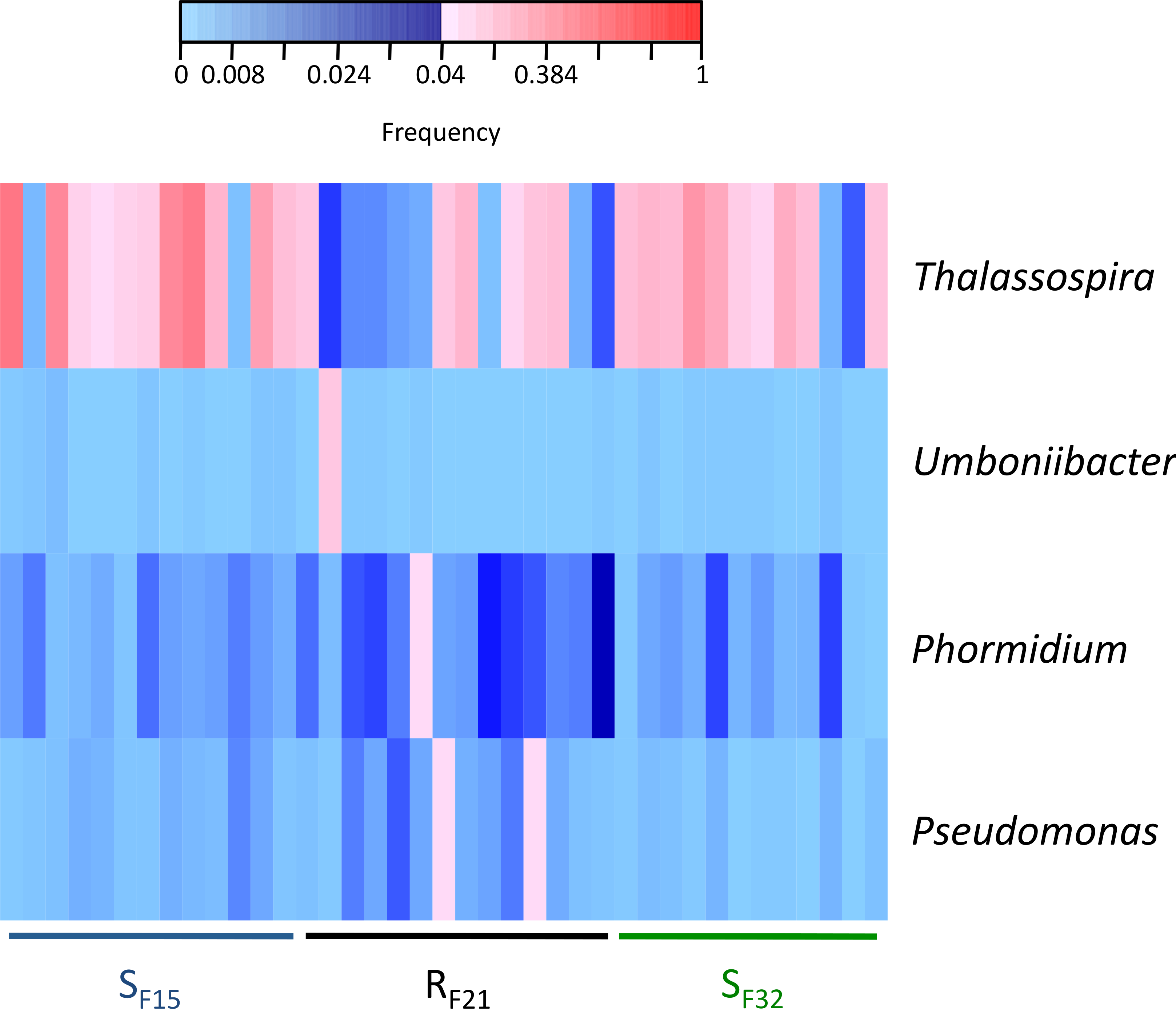

Supplement: Supplementary file 3 [file Image_3.TIFF]
